# Effects of Single Nucleotide Polymorphisms in the *SLC27A3* Gene on the Nutritional Value of Sheep Milk

**DOI:** 10.3390/ani10040562

**Published:** 2020-03-27

**Authors:** Ewa Pecka-Kiełb, Inga Kowalewska-Łuczak, Ewa Czerniawska-Piątkowska, Anna E. Zielak-Steciwko

**Affiliations:** 1 Department of Animal Physiology and Biostructure, Faculty of Veterinary Medicine, Wroclaw University of Environmental and Life Sciences, Norwida 31, 50-375 Wroclaw, Poland; 2 Department of Genetics, Faculty of Biotechnology and Animal Breeding, West Pomeranian University of Technology in Szczecin, Piastów Avenue 45, 79-311 Szczecin, Poland; inga.kowalewska-luczak@zput.edu.pl; 3 Department of Ruminant Science, Faculty of Biotechnology and Animal Husbandry, West Pomeranian University of Technology, ul. Klemensa Janickiego 29, Szczecin, 71-270 Szczecin, Poland; ewa.czerniawska-piatkowska@zut.edu.pl; 4 Institute of Animal Breeding Wroclaw University of Environmental and Life Sciences, ul. Chelmonskiego 38C, 51-631 Wroclaw, Poland; anna.zielak-steciwko@upwr.edu.pl

**Keywords:** sheep, milk, fatty acids, SNPs

## Abstract

**Simple Summary:**

The aim of the present study was to identify single nucleotide polymorphisms (SNPs) in the *SLC27A3* gene in sheep and to analyse correlations between selected genotypes and the nutritional value of milk. Milk and dairy products are basic elements of the human diet, due to their components having functional properties. Genetic potential of animals should be used to obtain food with the best technological and nutritional parameters. Results from the presented study suggest that milk from sheep with *TT* genotype at SNP4 is characterised by good technological and nutritional value. A high content of unsaturated fatty acids is observed in milk from sheep with *GG* in SNP1 and with *CC* in SNP3 of the *SLC27A3* gene.

**Abstract:**

The current research was undertaken to use the genetic potential of animals to obtain high-quality dairy products. Single nucleotide polymorphisms (SNPs) in *SLC27A3* gene were identified in Zošľachtená valaška sheep using polymerase chain reaction-restriction fragment length polymorphism (PCR-RFLP). Correlations between genotypes and milk composition and nutritional value were analysed This study showed that milk from sheep with *TT* genotype in the SNP4 locus was characterised by higher (*p* < 0.01) fat and dry matter content and lower lactose concentration, compared to sheep with *AA* and *TA* genotypes, respectively. Moreover, it was found that animals with *GG* genotype in SNP1 produced milk with higher C18:1n9c, C18:1n7t, CLA, and other unsaturated fatty acids (UFAs) content than sheep with *TT*. Additionally, milk from animals with *CC* at the SNP3 locus had significantly higher (*p* < 0.01) levels of UFAs than milk from sheep with other genotypes in the SNP3. In summary, it may be concluded that milk from animals with *TT* genotype of SNP4 is characterised by higher fat and dry matter content. Whereas, milk from sheep with *GG* in SNP1 and with *CC* in SNP3 is characterised by higher content of UFAs, which increases milk value as material for functional food production.

## 1. Introduction

Sheep milk is an excellent cheese-making material compared to cows’ milk, with its higher content of dry matter, total protein, crude fat, casein, and minerals and also contains more water-soluble vitamins [1]. An important parameter of sheep milk, which determines its calorific value, is fat content in the form of globules surrounded by phospholipid-protein membranes. The amount of fat in sheep milk is much higher comparing to milk produced by other livestock [2]. Furthermore, casein micelles contain more calcium than those in cow’s milk, and they are less hydrated and more thermally stable [3,4]. The profile of unsaturated fatty acids (UFAs) in milk determines the flavour and aroma of cheese [5]. Sheep milk is also a good source of unsaturated fatty acids, including conjugated linoleic acid (CLA). The levels of these fatty acids are much higher in sheep milk than in cow or goat milk, making it more desirable for consumers [6]. However, basic milk composition, protein fraction share and fatty acid profiles vary and depend on environmental and genetic factors [7].

The solute carrier 27A (*SLC27A*) gene, which consists of six members, encodes fatty acid transport proteins (FATPs). In the cell, these proteins may merge with cytomembranes and peroxisomal membranes. It has been demonstrated that FATPs 1-4 and -6 transport long- and very long-chain fatty acids, while FATP5 transports long-chain fatty acids and bile acids. FATPs may be found in both, cytomembranes and in the intracellular space, and they play a role in the absorption and activation of fatty acids [8]. In sheep, the *SLC27A3* gene (encoding FATP3) is located on chromosome 1, has 10 exons interspersed with introns, and encodes a protein made up of 680 amino acids.

So far, no data have been published on the correlation between the polymorphism in *SLC27A3* gene in sheep and the fatty acid profiles in milk. Additionally, little is known about the relation between milk quality traits and SNPs in the *SLC27A3* gene. Therefore, the aim of this study was to identify SNPs in the *SLC27A3* gene in Zošľachtená valaška sheep using PCR-RFLP and to analyse the relation between particular genotypes and milk technological quality nutritional value of milk.

## 2. Materials and Methods

### 2.1. Animals

Zošľachtená valaška sheep is a dairy breed that is a cross between local sheep and Texel, Hampshire, Cheviot, Leicester, and Lincoln sheep. These animals are well adapted to difficult mountain conditions. They are bred in Slovakia in the regions of Orava, Liptov, and Spiš at altitudes of over 800 m above sea level. Ewes usually weigh 50–55 kg, and rams 65–75 kg. Their wool is white with the length about 150 mm. Ewes are seasonally polyoestrous during the fall season (October–November). Prolificacy ranges from 110–130% and the twin pregnancy rate is low (2–15%). Once the lambs are weaned, the average milk yield is 80–120 kg during the 150 d of lactation.

The research was conducted on Zošľachtená valaška ewes (*n* = 50) in the first or second lactation during similar lactation phase (25–30 d). In the lambing period, the sheep were kept in special buildings complying with the European Union Directive standards (Journal of Laws 2010 No. 116, item 778) and were fed hay ad libitum, wheat middlings 250 g/ewe/day, and haylage 3 kg/ewe/day. In the purpose to collect milk samples the lambs were separated for one night from the mothers. Immediately after collection to sterile containers, samples were chilled at 4 °C. For DNA isolation, blood samples were collected from the external jugular vein in anticoagulant collection tubes containing K3EDTA. After transport to the laboratory all samples were frozen at −20 °C for subsequent analysis. The experimental procedures were done under veterinary care of University of Veterinary Medicine and Pharmacy in Košice, according to permit number IČO 00397474 2015, licensed by Ministry of Education, Sciences, Research and Sport of the Slovak Republic. 

### 2.2. SNP Detection and Genotyping

A MasterPure™ DNA Purification Kit for Blood Version II (Lucigen, Middleton, WI, USA) was used for DNA isolation according to manufacturer’s instructions. Single nucleotide substitutions selected for analyses are located on exons 2, 3, 4, and 7 of the *SLC27A3* gene and are responsible for missense mutations (Table 1). Genotyping of particular polymorphisms was performed using the PCR-RFLP method. Specific pairs of primers were designed using Primer3 program (http://bioinfo.ut.ee/primer3-0.4.0/) based on the *SLC27A3* gene DNA sequences from the Ensembl genome browser. Each PCR reaction (25 μL) contained 2 μL DNA, 1.0 μL forward primer, 1.0 μL of reverse primer, 12.5 μL 2× PCR Mix (A&A Biotechnology, Gdynia, Poland), and 8.5 μL nuclease-free water. Thermal conditions for the subsequent PCR stages were as follows: Initial denaturation at 94 °C for 5 min, followed by 30 cycles of denaturation at 94 °C for 30 s; annealing at a temperature specific for each primer pair for 45 s; elongation at 72 °C for 45 s; and final elongation at 72 °C for 5 min. PCR products were digested with restriction enzymes specific for each of the analysed polymorphisms. The restriction fragments were separated by electrophoresis on 3% agarose gel, stained with ethidium bromide and visualized on a UV transilluminator. Detailed information on the analysed SNPs—their location, amino acid substitution, primer sequences, annealing temperature, size of the PCR product, restriction enzymes and size of the fragments after restriction enzyme digestion are presented in Table 1.

### 2.3. Assessment of Technological Suitability of Milk

Content of milk fat, total protein, lactose, and dry matter were determined using an Infrared Milk Analyser 150 (Bentley Instruments Inc., Chaska, MN, USA), while urea level was measured by Chemspec (Bentley Instruments Inc.). Protein fraction shares, serum albumin, α-caseins, β-caseins, κ-caseins and α-lactalbumins, were determined using polyacrylamide gel electrophoresis in the presence of sodium dodecyl sulfate (SDS) according to the Laemmli’s method [9] as described previously by Pecka et al. [10].

The Folch method was used for milk fat extraction [11]. Fat was then converted into methyl esters of fatty acid using a 2 M KOH solution in methanol according to Christopherson and Glass [12]. Fatty acid profiles were determined using an Agilent Technologies 7890A gas chromatograph (Agilent Technologies, Santa Clara, CA, USA), with a flame ionization detector and HP-88 capillary column (100 cm length, 25 mm inner diameter, and 0.20 μm film thickness). The oven temperature was initially 50 °C and increased by 3 °C/min to 220 °C, while the detector and dispenser temperatures were −270 and 270 °C, respectively. Peaks were analysed by comparison with retention times of fatty acid methyl ester chromatographic patterns provided by Sigma using ChemStation software (Agilent Technologies).

### 2.4. Statistical Analysis

Statistical analysis of the association of single nucleotide polymorphisms and technological quality of sheep’s milk was carried out using Statistica 13.1 (StatSoft Poland, Krakow, Poland) and General Linear Model (GLM) software packages. The significance of differences between polymorphisms was determined with Tukey’s test. Hardy–Weinberg equilibrium and allele frequencies were calculated using PopGene version 1.32 software [13], and polymorphic information content (PIC) was established by applying the Nei and Roychoudhury method [14].

The statistical analysis was performed using the following model:

Yij = µ + ti + eij(1)

where Yij—analysed trait, µ—overall mean, ti—the effect of genotype on trait value, and eij—the effect of random error.

## 3. Results

In the analysed *SLC27A3* gene, three possible genotypes were identified for all examined polymorphic sites. Information concerning the frequency of genotypes and alleles for particular polymorphisms is presented in Table 2. For all analysed loci, only SNP1 deviated from Hardy-Weinberg equilibrium. PIC analysis indicated that all four substitutions, according to Botstein et al. [15] classification, showed average polymorphic information.

Basic milk composition and urea content in sheep milk in relation to particular genotypes of *SLC27A3* gene polymorphisms are presented in Table 3. Statistical analysis showed the influence (*p* < 0.01) of SNP substitution on the fat, lactose and dry matter content in sheep milk. In the case of SNP4 loci, it was demonstrated that milk from animals with *TT* genotype had higher (*p* < 0.01) content of fat and dry matter, but lower (*p* < 0.01) content of lactose compared to ewes with *AA*. Additionally, the milk from sheep with *AA* was characterised by higher (*p* < 0.05) levels of lactose compared to heterozygous animals.

Statistical analysis did not show any influence of the examined polymorphisms in the *SLC27A3* gene on the share of protein fractions in sheep milk (Table 4).

Relations between particular genotypes of the examined polymorphic loci with saturated fatty acids (SFAs) in sheep milk are presented in Table 5. Statistically significant differences (*p* < 0.01) related to analysed polymorphisms in the *SLC27A3* gene were found for several SFAs, including: caproic acid (C6:0), caprylic acid (C8:0), capric acid (C10:0), lauric acid (C12:0), and myristic acid (C14:0). Additionally, SNPs influenced (*p* < 0.05) the content of tridecanoic acid (C13:0), stearic acid (C18:0), and arachidic acid (C20:0). Analysis of the SNP1 polymorphism showed that the milk from heterozygotes was characterised by higher (*p* < 0.01) levels of eicosenoic acid (C20:0) compared to sheep with *GG*. The overall level of saturated fatty acids in sheep milk was higher (*p* < 0.01) in *TT* ewes than in animals with *GG* genotype. Additionally, the sum of saturated fatty acids was lower (*p* < 0.05) in sheep with *GG* than in heterozygous. In the case of the SNP2 polymorphism, milk from ewes with *GC* genotype was characterised by higher (*p* < 0.01) levels of C14:0 compared to sheep with *CC*. Analysis of the SNP3 polymorphism showed that ewes with *AA* genotype had higher (*p* < 0.01) content of C6:0, C8:0, C10:0, C12:0, and C14:0 in milk than animals with *CC*. Additionally, sheep with *AA* genotype had higher (*p* < 0.05) levels of C6:0 and C14:0 in milk than ewes with *AC*, while the level of C13:0 was higher (*p* < 0.05) in milk from sheep with *AA* compared to animals with *CC*. The milk produced by heterozygous animals was characterised by higher (*p* < 0.05) content of C12:0 than milk from ewes with *CC* genotype. The overall level of saturated fatty acids in milk sheep was higher (*p* < 0.01) in ewes with *AA* compared to animals with other genotypes in SNP3. In the case of the SNP4 polymorphism, the milk from ewes with *TT* had higher (*p* < 0.05) levels of C8:0 and C10:0 than milk from heterozygous.

Statistically significant differences (*p* < 0.01) related to analysed polymorphisms in the *SLC27A3* gene were found for several unsaturated fatty acids, including: myristic acid (C14:1), margaric-oleic acid (C17:1), cis 9 oleic acid (C18:1n9c), trans 7 oleic acid (C18:1n7t), linoleic acid (C18:2n6c), cis 9 trans 11 conjugated linoleic acid (CLA), and α-linolenic acid (C18:3n3) (Table 6). Analysis of the SNP1 polymorphism showed that the milk from sheep with *GG* was characterised by higher levels of C18:1n9c, C18:1n7t and CLA than the milk produced by animals with *TT*. Obtained results for SNP2 indicated significantly higher (*p* < 0.01) content of C18:2n6c and C18:3n3 and higher (*p* < 0.05) content of C18:1n7t in the milk from ewes with *CC* genotype compared to animals with *GG*. In comparison with heterozygous, higher (*p* < 0.01) levels of C18:2n6c was found in milk from ewes with *CC*. In the case of the SNP3 polymorphism, milk from sheep with *AA* had higher (*p* < 0.01) content of C14:1 and lower (*p* < 0.01) level of CLA compared to animals with *CC*. A similar relation was observed for C17:1 and C18:1n9c, ewes with *AA* had lower (*p* < 0.05) content of these fatty acids in milk than animals with *CC* genotype. The level of CLA in milk from ewes with *CC* was higher (*p* < 0.05) compared to heterozygous. The overall level of unsaturated fatty acids in sheep milk was considerably higher (*p* < 0.01) in animals with *CC* than in sheep with *AA* and *AC*. In heterozygous the UFAs content was higher (*p* < 0.05) than in milk from ewes with *AA*.

## 4. Discussion

Sheep milk is mainly used in cheese production. Fat and protein content in milk determines cheese yield. Moreover, basic milk composition is also a factor which determines the price of milk [16]. According to many authors, sheep milk contains 5.5–7.0% protein, 2.6–9% fat, 4.1–5.9% lactose, and 14.8–15.4% dry matter [17,18,19,20]. The results in the presented study are comparable with the values obtained by other authors.

This study demonstrated the considerable influence of the SNP4 polymorphism on the fat, lactose, and dry matter content in sheep milk. The milk from sheep with *TT* genotype had the highest content of fat, protein, and dry matter, whereas animals with *AA* produced milk with increased levels of lactose. Statistical analysis did not show any differences in urea levels among genotypes of all SNPs. This may be due to the fact that urea levels depend mainly on the content of crude protein and energy in animals’ diet [21]. So far, no data have been published describing the effect of polymorphisms analysed in present study on basic composition, shares of protein fractions and fatty acids profile in sheep milk.

Caseins are the main proteins in sheep milk, which are positively correlated with cheese production [2]. The levels of casein and whey fractions are affected, among others, by ambient temperature and animal diet [22]. Moreover, protein fraction shares are also determined by polymorphisms in genes, including: α-lactalbumin (*LALBA*), αS1-casein (*CSN1S1*), β-casein (*CSN2*), κ-casein (*CSN3*), β-lactoglobulin (*BLG*), annexin 9 (*ANXA9*), diacylglycerol O-acyltransferase (*DGAT1*) [19,23,24,25,26,27]. However, in the present study, the analysed polymorphisms, SNP1, SNP2, SNP3, and SNP4 in the *SLC27A3* gene, did not affect the profile of protein fractions in sheep milk.

The fatty acid profile of mammary gland secretion is characterised by a higher level of SFAs and a slightly lower level of UFAs [19,20]. The composition of milk fatty acids depends on breed, lactation stage, environment, and animals’ diet [10,27]. Moreover, fatty acid profile is affected by genetic factors, such as polymorphism of *BLG*, *DGAT1*, and Stearoyl-CoA Desaturase 1 (*SCD1*) genes [28,29,30].

Low-density lipoprotein (LDL) cholesterol has a negative impact on the cardiovascular system in humans. The level of SFAs in the human diet increases the level of the LDL cholesterol fraction in blood [31,32]. Saturated fatty acids that have the strongest influence on the increase in LDL cholesterol are C12:0 > C14:0 > C16:0 [33,34]. In the presented study, an increase in SFA levels in the milk from sheep with *TT* genotype in SNP1 and from sheep with *AA* in SNP3 was observed. Additionally, SNP3 *AA* ewes produced milk with higher levels of fatty acids from C6:0 to C14:0. Unsaturated fatty acids are desired in the human diet, and sheep milk is rich in them, in particular it contains a large amount of CLA. In milk, the level of SFAs decreases with the increase in UFAs [35]. In the present study, higher levels of CLA and other UFAs were found in homozygous SNP1 *GG* sheep and homozygous SNP3 *CC* animals, so the milk produced by these animals may be considered functional food with prevailing beneficial health effects for humans.

Furthermore, an increase in linoleic acid (C18:2n6c), which is a n-6 polyunsaturated fatty acid (PUFA), was observed in sheep with *CC* at SNP2 polymorphism, which accompanied an increase in α-linolenic acid (C18:3n3), which is a n-3 PUFA. No changes were observed in the levels of n-6 and n-3 fatty acids among genotypes of other polymorphisms. In the analysed samples, the ratio of n-6 and n-3 is constant. It is believed that food with high n-3 PUFA content and a low ratio of n-6 and n-3 has health promoting properties due to its anti-inflammatory capacities. Whereas, in humans, consumption of a diet containing a higher ratio of n-6:n-3 PUFAs results in chronic diseases, such as non-alcoholic fatty liver disease, cardiovascular diseases, inflammatory bowel disease, Alzheimer’s disease, obesity, and rheumatoid arthritis [36].

All the members of the fatty acids transport proteins (FATP) are membrane proteins with at least one transmembrane domain. Their N-terminus is positioned on the extracellular side of the membrane and the C-terminus on the cytosolic side. FATP family proteins have a highly conserved 311-amino acid sequence known as the AMP-binding protein domain located at the C-terminus, common to all adenylate-forming enzymes [8,37]. The product of the *SLC27A3* gene (FATP3) is responsible for long-chain fatty acids transport and very-long-chain fatty acids activation. It has been shown that FATP3 has acyl-CoA ligase activity. In the present study, sequence analysis of the FATP3 protein showed that the analysed substitutions contain sense-type mutations (missense), referring to amino acids that belong to the AMP-binding motif, not the fatty acids-binding motif (uniprot.org and smart.embl.de). 

Further analysis of the FATP3 protein sequence predicted that the changes in the amino acids resulting from SNP4 observed in *SLC27A3* gene probably have no effect on the function of this protein [38].

Previous research on the *SLC27A3* gene in sheep has concentrated on milk composition and milk technological quality [39,40], mainly due to its localisation on chromosome 1 (OAR1), close to regions rich in QTLs that determine milk yield and protein content [39]. Calvo et al. [39] showed correlation between analysed SNP and milk fat content, but authors did not observe correlation with milk technological quality. Considering the role of long-chain fatty acids as biologically-active compounds, it is likely that correlations between selected substitutions within the *SLC27A3* gene and various fatty acid fractions presented in this study result from the role of FATP3 as a transporter of long-chain fatty acids.

## 5. Conclusions

In the available literature, there are few studies on the correlation between selected genotypes and the nutritional value of sheep milk. The results presented in this study demonstrate that the milk produced by animals with *TT* genotype of SNP4 is characterised by a very good level of fat, protein, and dry matter. Examining sheep milk as a health-promoting food and looking for a desirable fatty acid profile, it was determined that milk produced by ewes with *GG* of SNP1 and with *CC* genotype of SNP3 had the highest UFAs content. In milk from sheep with *AA* genotype of SNP3, an elevated SFAs content was found, so their milk is less desirable in the human diet. However, our study is preliminary and should be repeated using other sheep breeds as well as a larger number of animals. 

## Figures and Tables

**Table 1:** Polymerase chain reaction-restriction fragment length polymorphism (PCR-RFLP) conditions for the analysed single nucleotide polymorphisms (SNPs) in the *SLC27A3* gene and information on the loci and substitution of amino acids.

SNP	*Loci*	Location/AA Change	Primer Sequence (5′–3′)	AT	AS	RE	PCR-RFLP Pattern (bp)
SNP1	*rs1090402056* c.754G > T	exon 2 Ala252Ser	F: GTAGAACTGCGGGGCTGTG R: AGGAGGTCATAGTTCCTGTTCC	53 °C	319	*Hpy*188 III	319/194, 125
SNP2	*rs600742549* c.958G > C	exon 3 Glu320Gln	F: GAGACAAGGCTTGGGTTCAG R: AGCCTCCTTCCTCTCCATTC	53 °C	354	*Scr*FI	222, 132/354
SNP3	*rs412479503* c.1096A > C	exon 4 Lys366Gln	F: TCTGGGAAGAAGGGAGTCAG R: TCTCCCCCTTCCATTTTCTT	50 °C	337	*Fnu4*HI	337/190, 147
SNP4	*rs593410192* c.1517T > A	exon 7 Val506Glu	F: CTCCAGGTTTGTGTCCAGGT R: TTTGGGTCCCAGAGATTCAG	51 °C	341	*Alu*I	179,162/ 164,162,15

AT—annealing temperature; AS—amplicon size; RE—restriction enzyme.

**Table 2:** Frequencies of genotypes and alleles of analysed polymorphisms in the *SLC27A3* gene.

Polymorphism	n	Genotype Frequencies		Allele Frequencies		χ^2^ (HWE)	PIC
SNP1	19 15 16	*GG* *GT* *TT*	0.38 0.30 0.32	*G* *T*	0.53 0.47	7.91 *	0.374
SNP2	15 23 12	*GG* *GC* *CC*	0.30 0.46 0.24	*G* *C*	0.53 0.47	0.294 ^ns^	0.374
SNP3	17 21 12	*AA* *AC* *CC*	0.34 0.42 0.24	*A* *C*	0.55 0.45	1.148 ^ns^	0.372
SNP4	15 22 13	*TT* *TA* *AA*	0.30 0.44 0.26	*T* *A*	0.52 0.48	0.703 ^ns^	0.375

n—number of animals; HWE—Hardy-Weinberg equilibrium; PIC—polymorphic information content; ns—non-significant; * *p* < 0.05.

**Table 3:** Milk composition and urea level from Zošľachtená valaška sheep in relation to particular genotypes of *SLC27A3* gene polymorphisms.

Parameter	SNP1			SNP2			SNP3			SNP4			SEM	*p*-Value
	GG	GT	TT	CC	GC	GG	AA	AC	CC	AA	TA	TT		
Fat ^1^	3.21	3.39	3.18	3.69	2.88	3.49	3.37	3.24	3.12	2.03 ^B^	3.05 ^B^	4.61 ^A^	1.107	<0.001
Protein ^1^	5.55	5.78	5.46	5.69	5.38 ^b^	5.83	5.73	5.54	5.47	5.35	5.34 ^b^	6.16 ^a^	0.738	0.053
Lactose ^1^	5.49	5.62	5.56	5.37	5.61	5.60	5.56	5.56	5.54	6.00 ^Aa^	5.51 ^b^	5.22 ^B^	0.407	0.001
DM ^1^	14.95	15.53	14.90	15.49	14.55	15.65	15.38	15.05	14.83	14.04 ^B^	14.59 ^B^	16.79 ^A^	1.525	<0.001
Urea ^2^	97.67	87.90	106.28	97.25	100.19	93.55	98.46	98.06	95.15	110.40	97.51	86.30	27.513	0.589

^1^ (%); DM: dry matter; ^2^ (mg × L^−1^); ^a,b^ values differ significantly between polymorphisms within rows (*p* < 0.05); ^A,B^ values differ highly significantly between polymorphisms within rows (*p* < 0.01).

**Table 4:** Percentage contribution of chosen protein fractions in milk from Zošľachtená valaška sheep in relation to particular genotypes of *SLC27A3* gene polymorphisms.

Protein Fractions	SNP1			SNP2			SNP3			SNP4			SEM	*p*-Value
	GG	GT	TT	CC	GC	GG	AA	AC	CC	AA	TA	TT		
Serum albumin (%)	14.26	14.08	13.16	13.06	14.48	13.52	13.86	14.32	13.03	13.70	14.69	12.76	3.426	0.846
α + β-casein (%)	43.65	43.90	43.25	45.17	42.13	44.59	44.11	44.24	45.25	44.11	43.03	43.98	6.252	0.947
κ-casein (%)	11.52	12.23	12.23	11.11	13.06	10.95	13.71	11.63	11.48	12.94	11.71	11.47	3.886	0.883
α-lactalbumin (%)	10.92	11.55	12.61	10.46	12.06	11.96	10.64	12.82	11.02	13.21	11.60	10.37	3.989	0.565

**Table 5:** Saturated fatty acids content in milk from Zošľachtená valaška sheep in relation to particular genotypes of *SLC27A3* gene polymorphisms.

Parameter	SNP1			SNP2			SNP3			SNP4			SEM	*p*-Value
	GG	GT	TT	CC	GC	GG	AA	AC	CC	AA	TA	TT		
C4:0 ^1^	0.58	0.63	0.59	0.67	0.55	0.60	0.72	0.55	0.48	0.54	0.56	0.70	0.226	0.124
C6:0 ^1^	0.73	0.77	0.82	0.81	0.73	0.80	0.93 ^Aa^	0.73 ^b^	0.61 ^B^	0.74	0.72	0.87	0.184	<0.001
C8:0 ^1^	0.88	0.97	0.98	0.94	0.91	0.98	1.09 ^A^	0.93	0.74 ^B^	0.92	0.86 ^b^	1.08 ^a^	0.195	<0.001
C10:0 ^1^	3.04	3.40	3.47	3.14	3.19	3.56	3.82 ^A^	3.28	2.54 ^B^	3.25	2.99 ^b^	3.75 ^a^	0.696	<0.001
C12:0 ^1^	2.07	2.25	2.27	2.08	2.15	2.33	2.46 ^A^	2.19 ^a^	1.79 ^Bb^	2.19	2.06	2.38	0.361	<0.001
C13:0 ^1^	0.04	0.05	0.06	0.05	0.05	0.05	0.06 ^a^	0.05	0.03 ^b^	0.05	0.04	0.06	0.023	0.026
C14:0 ^1^	7.63	7.78	8.05	7.27 ^B^	7.70 ^A^	8.41	8.55 ^Aa^	7.65 ^b^	7.04 ^B^	7.84	7.77	7.84	0.873	<0.001
C15:0 ^1^	1.03	1.09	1.13	1.12	1.07	1.08	1.15	1.09	0.97	1.06	1.05	1.15	0.161	0.105
C16:0 ^1^	21.81	21.60	22.22	21.63	21.78	22.22	22.49	21.77	21.19	21.61	21.80	22.22	1.188	0.190
C17:0 ^1^	1.00	1.08	1.01	1.07	1.03	0.99	1.02	1.03	1.03	1.05	1.03	0.99	0.110	0.359
C18:0 ^1^	10.97	12.47	12.38	11.81	12.11	11.54	11.97	11.95	11.58	12.27	12.22	11.01	1.474	0.032
C20:0 ^1^	0.29 ^B^	0.32 ^A^	0.35	0.32	0.32	0.31	0.34	0.32	0.28	0.31	0.32	0.32	0.048	0.014
ƩSFA	50.04 ^Bb^	52.69 ^a^	53.30 ^A^	51.11	51.64	52.86	54.59 ^A^	51.73 ^B^	48.31 ^C^	51.83	51.40	52.62	2.528	<0.001

SEM: standard error of the mean; ^1^ g/100 g of total fat concentration; SFAs: saturated fatty acids; ^a,b^ values differ significantly between polymorphisms within rows (*p* < 0.05); ^A,B^ values differ highly significantly between polymorphisms within rows (*p* < 0.01).

**Table 6:** Unsaturated fatty acids content in milk from Zošľachtená valaška sheep in relation to particular genotypes of *SLC27A3* gene polymorphisms.

Parameter	SNP1			SNP2			SNP3			SNP4			SEM	*p*-Value
	GG	GT	TT	CC	GC	GG	AA	AC	CC	AA	TA	TT		
C14:1 ^1^	0.54	0.56	0.60	0.57	0.55	0.58	0.62 ^A^	0.56	0.49 ^B^	0.52	0.56	0.62	0.091	<0.001
C16:1 ^1^	5.66	5.40	5.83	5.38	5.68	5.78	5.37	5.69	5.92	5.39	5.87	5.51	0.676	0.157
C17:1 ^1^	0.53	0.48	0.48	0.51	0.51	0.48	0.46 ^b^	0.51	0.54 ^a^	0.51	0.51	0.48	0.060	0.002
C18:1n9c ^1^	23.64 ^A^	22.45	21.21 ^B^	22.95	22.33	22.41	21.27 ^b^	22.45	24.34 ^a^	23.00	22.30	22.36	1.979	<0.001
C18:1n9t ^1^	1.75	1.87	2.00	1.74	1.96	1.83	1.68	1.92	2.04	1.86	2.01	1.66	0.399	0.050
C18:1n7t ^1^	2.25 ^A^	2.16	1.92 ^B^	2.26 ^a^	2.14	1.96 ^b^	2.02	2.09	2.31	2.21	2.10	2.06	0.258	<0.001
C18:2n6c ^1^	1.87	1.77	1.68	2.14 ^A^	1.77 ^Ba^	1.50 ^Bb^	1.66	1.85	1.82	1.78	1.76	1.80	0.261	<0.001
CLA ^1^	1.46 ^A^	1.19 ^B^	1.00 ^B^	1.33	1.24	1.14	1.13 ^B^	1.20 ^b^	1.42 ^Aa^	1.26	1.22	1.22	0.215	<0.001
C18:3n3 ^1^	1.54	1.48	1.48	1.71 ^A^	1.50	1.35 ^B^	1.43	1.56	1.51	1.53	1.50	1.49	0.201	0.004
C20:1 ^1^	0.08	0.07	0.07	0.08	0.07	0.07	0.07	0.08	0.06	0.08	0.07	0.07	0.028	0.816
C20:4n6 ^1^	0.10	0.10	0.10	0.12	0.10	0.09	0.10	0.11	0.09	0.10	0.09	0.11	0.031	0.134
EPA ^1^	0.07	0.09	0.08	0.09	0.09	0.07	0.08	0.09	0.08	0.08	0.08	0.08	0.024	0.608
ƩUFA	40.48 ^A^	38.42	37.22 ^B^	39.74	38.87	38.00	36.65 ^Bb^	38.90 ^Ba^	41.74 ^A^	39.07	38.89	38.49	2.294	<0.001

SEM: standard error of the mean; ^1^ g/100 g of total fat concentration; UFAs: unsaturated fatty acids ^a,b^ values differ significantly between polymorphisms within rows (*p* < 0.05); ^A,B^ values differ highly significantly between polymorphisms within rows (*p* < 0.01).
